# CyTOF analysis of immune characteristics in cSLE: belimumab treatment and refractory cases

**DOI:** 10.3389/fimmu.2026.1699104

**Published:** 2026-02-02

**Authors:** Ying Luo, Linlin Wang, Yongbin Xu, Qian Chen, Ki Pui Lam, Xiaona Zhu, Qiru Su, Shuli Luo, Jun Yang

**Affiliations:** 1 Department of Rheumatology and Immunology, Shenzhen Children’s Hospital, Shenzhen, China; 2 Division of Immunology, Boston Children’s Hospital, Boston, MA, United States; 3 Department of Pediatrics, Harvard Medical School, Boston, MA, United States; 4 Clinical Research Unit, Shenzhen Children’s Hospital, Shenzhen, China

**Keywords:** biologics, BLys, CD38, CD73, childhood-onset systemic lupus erythematosus

## Abstract

**Introduction:**

Systemic lupus erythematosus (SLE) is a chronic autoimmune disease affecting multiple organs. Some patients still develop severe refractory SLE despite conventional treatments. Belimumab, a biologic targeting B lymphocyte stimulator (BLyS), shows therapeutic promise in SLE, but its effects on pediatric patients remain unclear.

**Methods:**

This study used CyTOF to analyze immunophenotypic changes before and after belimumab treatment and investigate immune features in refractory SLE.

**Results:**

Results showed that belimumab treatment primarily reduced transitional and naïve B cells, and also decreased age-associated B cells (ABCs), a subset implicated in autoimmunity or chronic inflammation. Plasma cells and plasmablasts persisted in refractory cases. CD38 expression on B cells was elevated in severe disease and correlated with disease activity, while CD73 expression increased during recovery and inversely correlated with activity.

**Discussion:**

These findings indicate that combining belimumab with conventional therapy induces significant immune modulation, particularly within B cells. Persistent plasma cells and plasmablasts may contribute to disease refractoriness. CD38 and the CD73-mediated adenosine pathway may provide insights for future research strategies in SLE.

## Graphical Abstract

**Figure:**
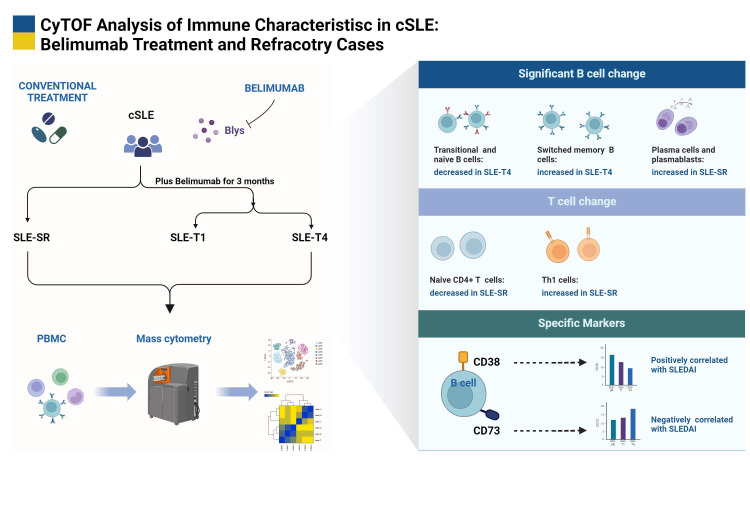
Using high-dimensional CyTOF, this study characterized immune cell dynamics in childhood-onset SLE (cSLE) during belimumab treatment. Belimumab primarily led to a reduction in transitional and naïve B cells. Plasma cells and plasmablasts persisted in refractory patients, highlighting CD38 as a feature of interest. CD73 upregulation on B cells correlated with reduced disease activity, suggesting a potential role for the CD73-mediated adenosine pathway in immune modulation. Childhood-onset systemic lupus erythematosus, Biologics, BlyS, CD38, CD73.

## 1 Introduction

Systemic lupus erythematosus (SLE) is a complex multisystem autoimmune disease characterized by dysregulation in innate and adaptive immune systems, marked by producing numerous autoantibodies (1). Childhood-onset systemic lupus erythematosus (cSLE) typically presents with a more aggressive course with higher flare rates and mortality, especially during initial diagnosis and follow-up (2). Additionally, cSLE patients have unique growth and developmental needs, requiring safer and less toxic treatment options. Recent advances in SLE treatment, particularly with targeted therapies and biologics like belimumab, have shown promise (3, 4). However, some SLE patients, including children, remain unresponsive to these treatments and are classified as refractory (5, 6).

Belimumab is the only biologic-targeting medicine approved for treating systemic lupus erythematosus (SLE) in adults and children in nearly 50 years (7–9). It is a specific inhibitor of B lymphocyte stimulator (BLyS). By blocking the binding of BLyS to B cell surface receptors, belimumab inhibits the survival, proliferation, and differentiation of B cells, thereby reducing the production of autoantibodies. BLyS can bind to three known receptors: transmembrane activator and CAML interactor (TACI), B cell maturation antigen (BCMA), and BAFF receptor (BAFF-R or BR3). BLyS has the highest affinity for BR3, which is primarily expressed in B cells as they migrate from the bone marrow to the periphery circulation and develop into mature B cells, making these cells the primary target of belimumab (10). Emerging evidence also suggests that BR3 may be expressed on regulatory T cells and activated T cells; however, the effects of belimumab on these cells remain incompletely understood (11, 12).

Therefore, this study aimed to characterize immune phenotypic differences among cSLE patients with distinct clinical statuses and to evaluate the effects of belimumab on circulating immune cell populations. Additionally, the immunophenotype of refractory cSLE patients was profiled descriptively as a reference for identifying potential therapeutic targets.

## 2 Materials and methods

### 2.1 Study subjects

Fifteen pediatric patients with SLE were enrolled at Shenzhen Children’s Hospital between July 2021 and October 2022. The diagnosis of SLE was made according to the 1997 American College of Rheumatology (ACR) criteria and/or the 2019 European League Against Rheumatism (EULAR)/ACR classification criteria. Patients were eligible if they had received at least one month of standard immunosuppressive therapy (mycophenolate mofetil (MMF) and prednisone, with or without hydroxychloroquine). Seven patients had previously received cyclophosphamide (CTX) and were transitioned to MMF for maintenance, while the remaining eight were newly diagnosed and initiated MMF as part of induction therapy. Exclusion criteria included active infection or malignancy, confirmed monogenic or genetic forms of SLE, and known hypersensitivity to belimumab or its components.

Belimumab was administered intravenously at weeks 0, 2, and 4, followed by maintenance infusions every four weeks. Clinical assessments were performed at three time points: baseline (SLE-T0), immediately before the first belimumab infusion (SLE-T1), and three months after treatment initiation (SLE-T4). Peripheral blood samples for immunological analysis were collected at SLE-T1 and SLE-T4.

In addition, three pediatric patients with severe refractory SLE (SLE-SR) were included as an independent, descriptive reference group. Refractory disease was operationally defined as: disease duration exceeding three years, prior exposure to more than three immunosuppressive or biologic agents, failure to achieve sustained clinical remission, persistent significant proteinuria (>1g/24 h) despite at least six months of lupus nephritis therapy, and an SLE Damage Index (SDI) score ≥ 1. Given their long-standing disease course, heterogeneous organ involvement, and prior exposure to biologics, the SLE-SR cohort was not intended for direct statistical comparison with the longitudinal cohort, but served as a descriptive reference for immune phenotypes.

The study protocol was approved by the local institutional ethics committee, and written informed consent was obtained from all participants according to the Declaration of Helsinki.

### 2.2 Clinical evaluation

SLE disease activity was assessed using the SLE Disease Activity Index 2000 (SLEDAI-2K) and the Physician’s Global Assessment (PGA). The SLE Disease Damage Index (SDI) was used to evaluate accumulated organ damage. Low disease activity was defined following the Lupus Low Disease Activity State (LLDAS) criteria, including a SLEDAI-2K score of ≤ 4, no active disease in major organ systems, no new SLE activity compared to previous assessments, a PGA score ≤ 1, a current prednisone-equivalent dose ≤ 7.5 mg/day, and stable maintenance therapy with immunosuppressants or approved biologic agents (13).

### 2.3 CyTOF analysis of PBMCs

Peripheral blood mononuclear cells (PBMCs) were isolated from peripheral blood by density gradient centrifugation. Cells were stained for viability using cisplatin and incubated with an Fc receptor–blocking solution to minimize non-specific binding. Surface and intracellular markers were stained using standardized antibody panels, and mass-tag cellular barcoding (MCB) was applied to reduce variability in staining efficiency and instrument sensitivity (14). Details of all 43 metal-conjugated antibodies, including target antigen, clone, and metal tag, are provided in **Supplementary Table S1**.

Data acquisition was performed on a Helios CyTOF system (Fluidigm) with EQ calibration beads, and raw FCS files were normalized using bead-based normalization (15). Acquisition order was randomized to minimize batch effects. Data preprocessing included removal of debris, doublets, and dead cells. For visualization, 30,000 cells were randomly downsampled from each donor sample and analyzed using t-SNE (perplexity = 100, iterations = 1000). Unsupervised clustering was performed using the PhenoGraph algorithm, and hierarchical clustering of median marker expression was applied to assess cluster consistency across samples (16). Clusters were annotated based on canonical lineage markers and subset-specific marker expression, and mapped to major immune cell populations. Marker definitions and cluster annotations for each immune cell subset are provided in the supplementary Excel (Supplementary_Table_Immune_Cell_Subsets_CyTOF.xlsx). The full CyTOF experimental procedures—including sample preparation, staining, data acquisition, preprocessing, dimensionality reduction, clustering parameters, and annotation criteria—are detailed in the **Supplementary Methods** for CyTOF Analysis.

### 2.4 Statistical analysis

Statistical analyses were performed using GraphPad Prism 10.0 for basic comparisons and R (version 4.3.0) for high-dimensional data analyses. For the longitudinal cSLE cohort, each patient served as their own control, and paired t-tests or Wilcoxon matched-pairs tests were used to assess changes between time points (SLE-T1 vs. SLE-T4), depending on data distribution. For comparisons involving the SLE-SR cohort, which served as an independent descriptive reference group, no formal statistical testing was performed.

For CyTOF high-dimensional cluster analyses, donor-level statistics were applied. Multiple testing across clusters was controlled using the Benjamini-Hochberg false discovery rate (FDR) method, and FDR-adjusted p-values (q-values) are reported where applicable. Multivariate correlation analysis was used to assess correlations between immune phenotypes and clinical parameters. Data are presented as mean ± standard error of the mean (SEM), unless otherwise specified. Statistical significance was defined as P < 0.05 or q < 0.05 after FDR correction.

## 3 Results

### 3.1 Clinical characteristics of cSLE cohorts

Fifteen pediatric patients with cSLE were followed longitudinally at three time points: baseline (SLE-T0), immediately prior to belimumab initiation after at least one month of standard therapy (SLE-T1), and three months after treatment initiation (SLE-T4). Baseline characteristics of the longitudinal cohort are summarized in **Table 1**, and longitudinal clinical data across T0, T1, and T4 are presented in **Table 2**. Adjunctive belimumab therapy for three months led to notable improvements, including reductions in SLEDAI-2K scores to below 4, normalization of complement levels, and tapering of prednisone to one-quarter of the baseline dose. Most patients achieved low disease activity by the end of the study.

**Table 1:** Baseline demographic, clinical, and laboratory characteristics of cSLE patients prior to belimumab therapy (SLE-T0).

Characteristics	n (%)
Demographic features	
Female	15 (100%)
Age, months^#^	119 (70-190)
Disease duration, months^#^	5 (1-14)
SLE manifestations	
Cutaneous manifestations (malar rash, and/or discoid rash, oral ulcers)	10 (67%)
Articular involvement (arthritis)	6 (40%)
Renal involvement	11 (73%)
Hematological involvement	8 (53%)
NPSLE	0
Serositis (pulmonary/pericardic effusion)	3 (21%)
Antiphospholipid Syndrome	3 (21%)
SLEDAI 2K-score^#^	15 (8-29)
SDI	0
PGA	2 (1-3)
Laboratory parameters	
Reduced serum levels of C3 and/or C4	14 (93%)
Anti-dsDNA positivity	15 (100%)
aPL positivity	5 (37%)
Treatment	
Steroids (prednisone)	15 (100%)
Dosage of prednisone (mg/kg/d) ^#^	1.25 (0.56-1.88)
Use of hydroxychloroquine	14 (93%)
Use of immunosuppressive drugs*	15 (100%)

**^#^**Data are expressed as median (min-max).

*7 patients Use of CTX+MMF (sequential therapy), 8 patients Use of MMF.

CTX: Cyclophosphamide; MMF: Mycophenolate Mofetil; NPSLE: neuropsychiatric SLE; SLEDAI-2K score: SLE Disease Activity Index 2000; SDI: SLE International Collaborating Clinics/American College of Rheumatology Damage Index; PGA: Physician’s Global Assessment; dsDNA: double-stranded DNA; aPL: Antiphospholipid Antibodies.

**Table 2:** Clinical and laboratory profiles of cSLE patients across belimumab treatment (SLE-T0, SLE-T1, and SLE-T4).

Parameters	T0 (n=15)	T1 (n=15)	T4 (n=15)	Friedman’s ANOVA on ranks (*p-value*)
Disease activity				
SLEDAI 2K-score	15.1 (5.6)	8.3 (4.3)	3.5 (3.1)	<0.001
Laboratory parameters (normal values)				
Serum levels of C3 (g/L) (0.8-1.6)	0.36 (0.06)	0.817 (0.08)	0.98 (0.08)	<0.001
Serum levels of C4 (g/L) (0.1-0.4)	0.05 (0.01)	0.09 (0.01)	0.15 (0.02)	<0.001
Serum levels of anti-dsDNA (IU/ml) (<100)	543.2 (85.3)	252.8 (51.9)	157.0 (42.8)	<0.001
ESR (mm/h) (<20)	31.2 (25.0)	13.8 (12.9)	8.6 (8.4)	0.002
Treatment				
Dosage of prednisone (mg/kg/d)	1.25 (0.62)	0.70 (0.60)	0.33 (0.19)	<0.001

Data are expressed as mean (standard deviation).

T0, untreated; T1, before Belimumab treatment; T4, 3 months after Belimumab treatment.

SLEDAI-2K score, SLE Disease Activity Index 2000; dsDNA, double-stranded DNA; ESR, Erythrocyte Sedimentation Rate.

Three additional pediatric patients with severe refractory SLE (SLE-SR) were included as an independent reference group, representing a clinically distinct population with long-standing disease, multi-organ involvement, extensive prior therapy, and persistent disease activity. Clinical characteristics of the SLE-SR patients are summarized in **Table 3**, including disease duration, organ involvement, SDI, disease activity scores, and prior/current immunosuppressive regimens. This cohort exhibited substantial heterogeneity, reflecting the complexity of refractory cSLE. Analyses involving SLE-SR were limited to descriptive immunophenotypic presentation, without formal statistical comparison to the longitudinal cohort.

**Table 3:** Historical and current clinical manifestations and treatments in severe refractory SLE (SLE-SR). .

Characteristics	Patient 1	Patient 2	Patient 3
Age, sex	15.5y, female	15.3y, female	16y, female
Diagnosis of SLE(year)	2018	2018	2018
Clinical manifestations, history	Lupus-nephritis II, fever, autoimmune hemolytic anemia, severe anemia, lupus carditis, incomplete intestinal obstruction, sinus bradycardia, skin rash	Lupus-nephritis II, skin rash, oral ulcer, arthritis, anticardiolipin syndrome	Lupus-nephritis IV-S(A)+V, NPSLE, convulsive, hyperthyroidism
Clinical manifestations, current	Lupus nephritis, autoimmune hemolytic anemia, lupus carditis	Lupus nephritis, anticardiolipin syndrome	NPSLE, lupus nephritis, diabetic
SDI	1	1	1
SLEDAI 2K-score	14	12	8
SLE treatment, history	CTX (accumulated dose 160mg/kg), MMF, prednisolone, hydroxychloroquine, belimumab, tacrolimus, IVIG	CTX(accumulated dose 160mg/kg), MMF, prednisolone, hydroxychloroquine, cyclosporin A, rituximab, belimumab, tacrolimus, IVIG	CTX (accumulated dose 120mg/kg), MMF, prednisolone, hydroxychloroquine, cyclosporin A, belimumab, tacrolimus, IVIG
SLE treatment, current	Prednisolone 40 mg daily, hydroxychloroquine 0.15 g daily, CTX (second round)	Prednisolone 50 mg daily, tacrolimus 3.5 mg daily, hydroxychloroquine 0.2g daily	Prednisolone 17.5 mg daily, tacrolimus 4 mg daily, hydroxychloroquine 0.2g daily

SLE-SR, severe refractory SLE; CTX, Cyclophosphamide; MMF, Mycophenolate Mofetil; NPSLE, neuropsychiatric SLE; SLEDAI-2K score, SLE Disease Activity Index 2000; SDI, SLE Damage Index; IVIG, Intravenous Immunoglobulin.

**Figure 1:**
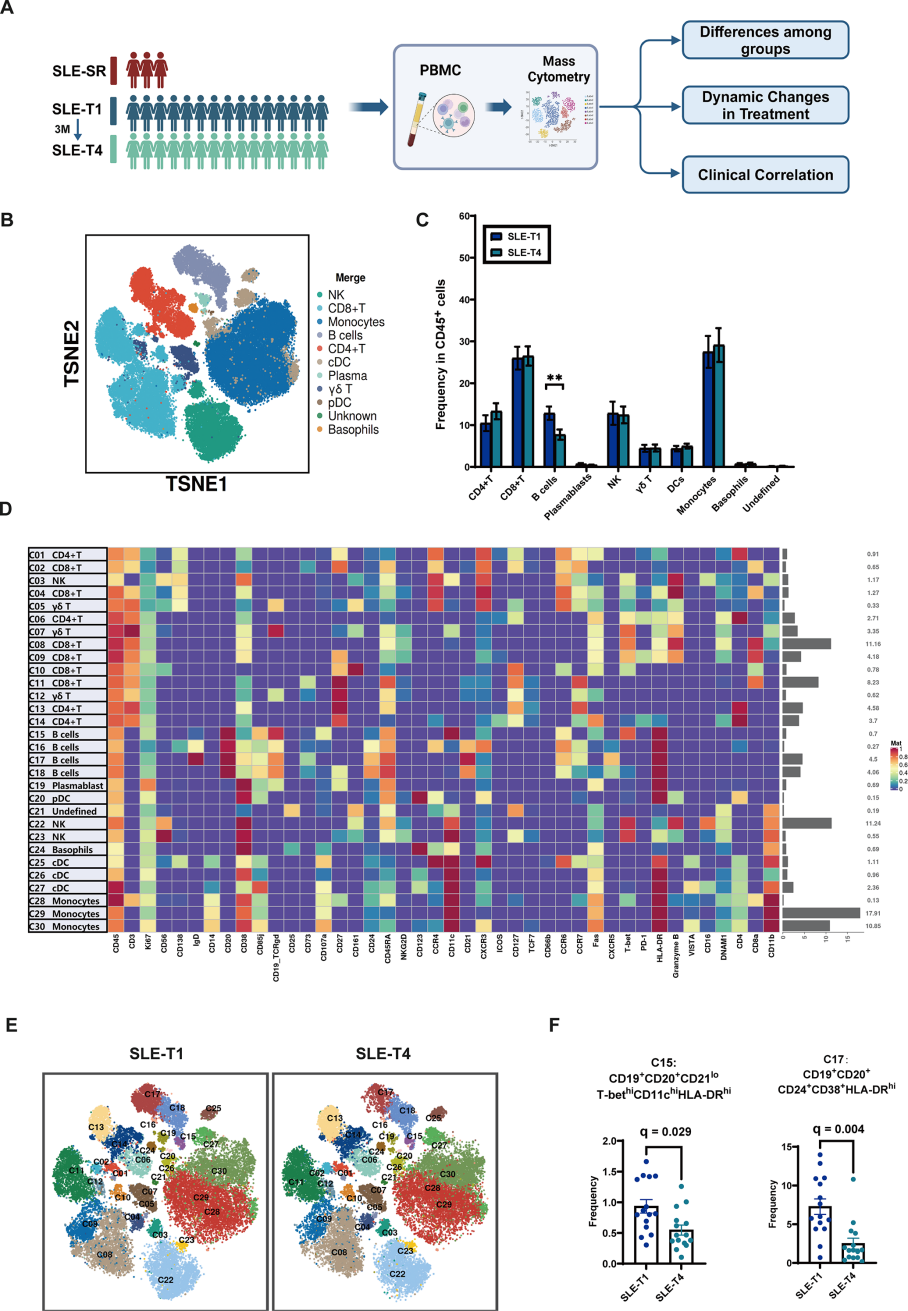
Visualization and clustering of PBMC in cSLE. **(A)** Study design. The main longitudinal cohort (n = 15) underwent clinical assessments at baseline (SLE-T0), pre-belimumab (SLE-T1), and 3 months post-treatment (SLE-T4). Peripheral blood samples for immune profiling were collected at SLE-T1 and SLE-T4. Three pediatric patients with severe refractory SLE (SLE-SR) were included as an independent, descriptive reference group. **(B)** t-SNE visualization and cell phenotype categorization of PBMCs. **(C)** Paired analysis of PBMC subpopulation changes between SLE-T1 and SLE-T4. **(D)** Heatmap showing scaled expression of lineage-defining marker genes across identified cell clusters. Each row represents a cell cluster, and each column represents a marker. **(E)** t-SNE distributions of immune cell clusters at SLE-T1 and SLE-T4. **(F)** Paired comparison of PBMC subclusters between SLE-T1 and SLE-T4. Donor-level statistics were applied for CyTOF cluster analyses. PBMC, peripheral blood mononuclear cells; NK, Natural Killer cells; pDC, Plasmacytoid Dendritic Cells; cDC, Conventional Dendritic Cells; HLA-DR, Human Leukocyte Antigen-DR; T-bet, T-box transcription factor TBX21. Each symbol represents one subject. Error bars represent the standard error of the mean (SEM). Statistical significance for P values is indicated as: *P < 0.05, **P < 0.01. FDR-adjusted p-values (q-values) are reported as exact numbers and displayed in the figure where applicable.

PBMC immune phenotypes were then analyzed across all three groups to characterize immunophenotypic differences, assess dynamic changes with belimumab treatment, explore correlations with clinical parameters, and descriptively highlight specific cell subpopulations or surface markers. The SLE-SR cohort served as a reference for refractory immune profiles, and the overall study design and immune analysis framework are summarized in the schematic diagram (**Figure 1A**).

### 3.2 Visualization and clustering of PBMC between SLE-T1 and T4

As a first step, unsupervised clustering was performed to identify immune cell subpopulations, and t-SNE was applied to visualize the peripheral blood immune landscape. Based on canonical cell surface markers, PBMCs were classified into major immune lineages, including T cells, B cells, dendritic cells (DCs), monocytes, plasma cells, natural killer (NK) cells, and basophils (**Figure 1B****).**

Paired analyses between SLE-T1 and SLE-T4 demonstrated a significant reduction in overall B cell frequency, whereas no significant changes were observed in other major immune cell compartments (**Figure 1C**).

Further unsupervised clustering identified 30 distinct immune cell subclusters. By examining marker expression patterns in the heatmap, each cluster was annotated to a corresponding immune cell type, illustrating the relationships between clusters and defining markers (**Figure 1D****).** The t-SNE distributions of these clusters at SLE-T1 and SLE-T4 are shown in **Figure 1E**. In paired analyses, C15 (CD19^+^CD20^+^CD21^lo^T-bet^hi^CD11c^hi^HLA-DR^hi^), known as age-associated B cells (ABCs) and linked with autoimmunity or chronic inflammation, demonstrated a significant reduction following treatment (17). Cluster C17, a transitional B cell populations (CD19^+^CD20^+^CD24^+^CD38^+^HLA-DR^hi^), also showed significant reductions at SLE-T4 compared with SLE-T1 (**Figure 1F**).

### 3.3 B-cell analysis between SLE-T1 and T4

**Figure 2:**
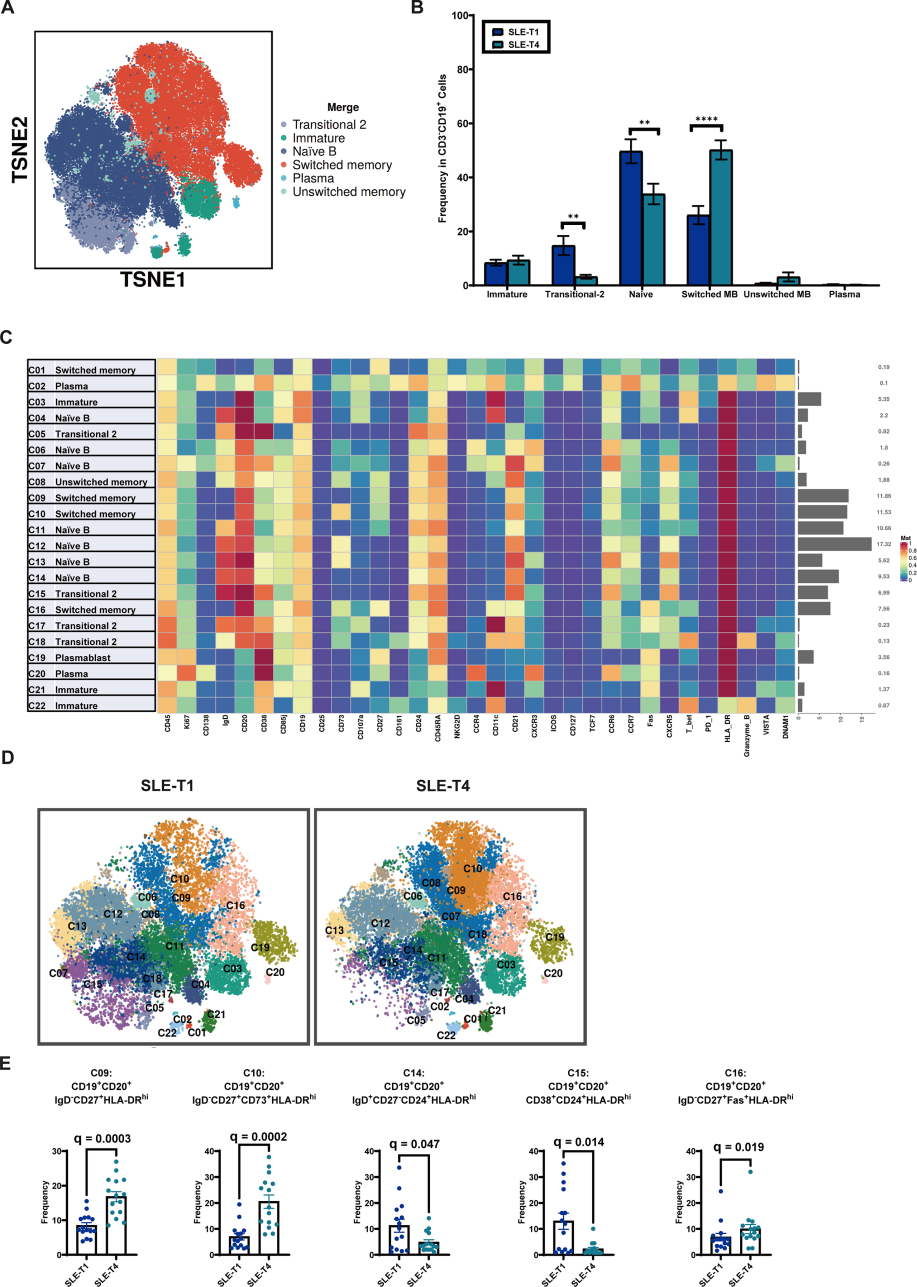
Characterization of B-cell phenotypes and subpopulations. **(A)** t-SNE visualization of B cells and their phenotypic categorization. **(B)** Paired comparison of B-cell subpopulation proportions between SLE-T1 and T4. **(C)** Heatmap showing median expression of lineage and functional markers across 22 B-cell clusters. **(D)** t-SNE visualization of B-cell clusters at SLE-T1 and T4. **(E)** Paired comparison of B-cell cluster proportions between SLE-T1 and T4. MB, Memory B Cells; HLA-DR, Human Leukocyte Antigen-DR; FAS, Fas Receptor. Each symbol represents one subject. Error bars represent the standard error of the mean (SEM). Significance levels are indicated as: *P *<* 0.05, **P < 0.01, ***P *<* 0.001, ****P *<* 0.0001. FDR-adjusted p-values (q-values) are reported as exact numbers and displayed in the figure where applicable.

Following the global PBMC analysis, we next focused on B-cell phenotypes and their dynamic changes between SLE-T1 and SLE-T4. Using core B-cell markers, including CD19, CD20, IgD, CD27, CD24, and CD38, B cells were classified into six major phenotypic subpopulations: naïve B cells, immature B cells, transitional-2 B cells, switched memory B cells (switched MB), unswitched memory B cells (unswitched MB), and plasma cells (**Figure 2A**).

Paired comparisons between SLE-T1 and SLE-T4 revealed marked reductions in the proportions of naïve B cells and transitional-2 B cells, accompanied by an increase in switched MB cells, whereas no significant change was observed in plasma cell frequencies (**Figure 2B**).

To further resolve B-cell heterogeneity at the cluster level, we applied the PhenoGraph algorithm using 33 markers for unsupervised re-clustering of B cells, identifying 22 distinct B-cell clusters. A heatmap depicting the median expression levels of lineage and functional markers across these clusters illustrates cluster-specific expression patterns and facilitated biological annotation of each cluster (**Figure 2C****).**

The t-SNE plots illustrate the distribution of B-cell clusters at SLE-T1 and SLE-T4 (**Figure 2D**). At the cluster level, paired analysis showed that multiple clusters corresponding to switched MB cells (C09, C10, and C16) exhibited increased proportions at SLE-T4, whereas clusters corresponding to naïve B cells (C14) and transitional B cells (C15) were reduced compared with SLE-T1 (**Figure 2E****).** Notably, most B-cell clusters exhibited high expression of HLA-DR, indicating a generally activated B-cell state.

### 3.4 T-cell and other cell subsets analysis between SLE-T1 and T4

**Figure 3:**
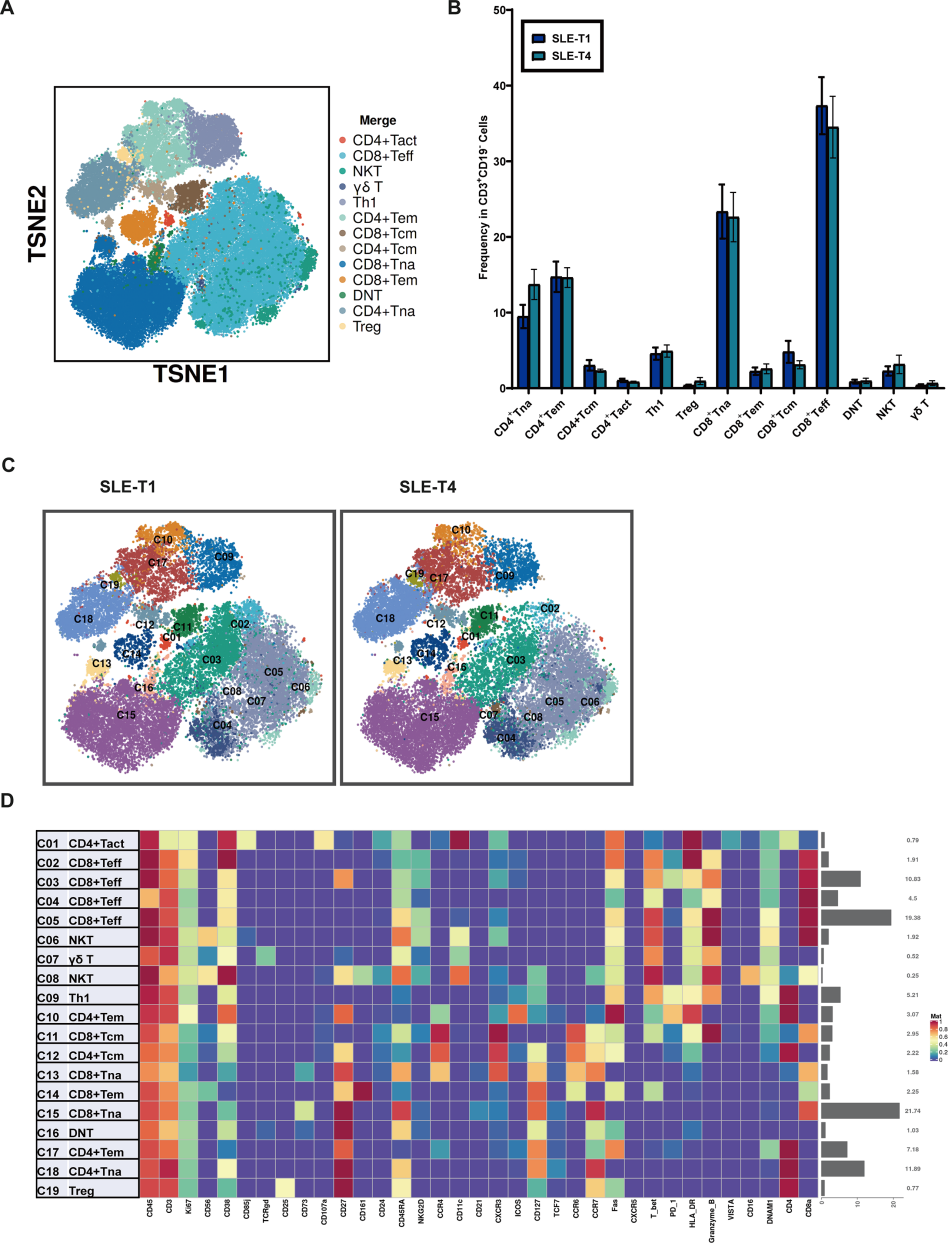
Characterization of T Cell expression patterns and subpopulations. **(A)** t-SNE visualization of T cells and their phenotypic categorization. **(B)** Paired comparison of T-cell subpopulation proportions between SLE-T1 and T4. **(C)** t-SNE visualization of T-cell clusters at SLE-T1 and T4. **(D)** Heatmap showing median expression of lineage and functional markers across 19 T-cell clusters. Tna, Naive T cells; Tem, Effector Memory T cells; Tcm, Central Memory T cells; Tact, Activated T cells; Th, Helper T cells; Treg, Regulatory T cells; Teff, Effector T cells; DNT, Double Negative T cells; NKT, Natural Killer T cells. Error bars represent the standard error of the mean (SEM).

Subsequently, we conducted an in-depth analysis of T-cell populations between SLE-T1 and T4. The identified T-cell subpopulations included activated CD4 T cells, naïve CD4 T cells, effector CD4 T cells, central memory CD4 T cells, regulatory T cells (Tregs), Th1 cells, naïve CD8 T cells, effector memory CD8 T cells, central memory CD8 T cells, natural killer T cells (NKT), and γδ T cells (**Figure 3A****).** Paired comparisons between SLE-T1 and T4 showed no significant changes in the proportions of these T-cell subpopulations (**Figure 3B**).

To further resolve T-cell heterogeneity, we performed re-clustering using 35 markers and identified 19 distinct T-cell clusters (**Figure 3C**). A heatmap illustrating the expression patterns of lineage and functional markers across all T-cell clusters is shown in **Figure 3D**. Paired cluster-level analysis revealed no significant changes in any of these clusters between SLE-T1 and T4.

We also analyzed myeloid and innate immune cell populations. Canonical subpopulations, including plasmacytoid dendritic cells (pDCs), conventional dendritic cells (cDCs), basophils, monocytes, and natural killer (NK) cells, were identified, and paired comparisons revealed no significant differences in their proportions between SLE-T1 and T4. Further re-clustering of CD3^−^CD19^−^ immune cells identified 21 distinct subclusters; none of these clusters showed significant changes (**Supplementary Figure S1****).**

### 3.5 Expression profiling and correlation analysis of specific markers

After comparing changes in immune cell subpopulations between SLE-T1 and SLE-T4, we further examined the expression profiles of several functional surface markers within the B-cell compartment. Among these, CD38 and CD73 showed pronounced alterations and were therefore selected for focused analysis.

**Figure 4:**
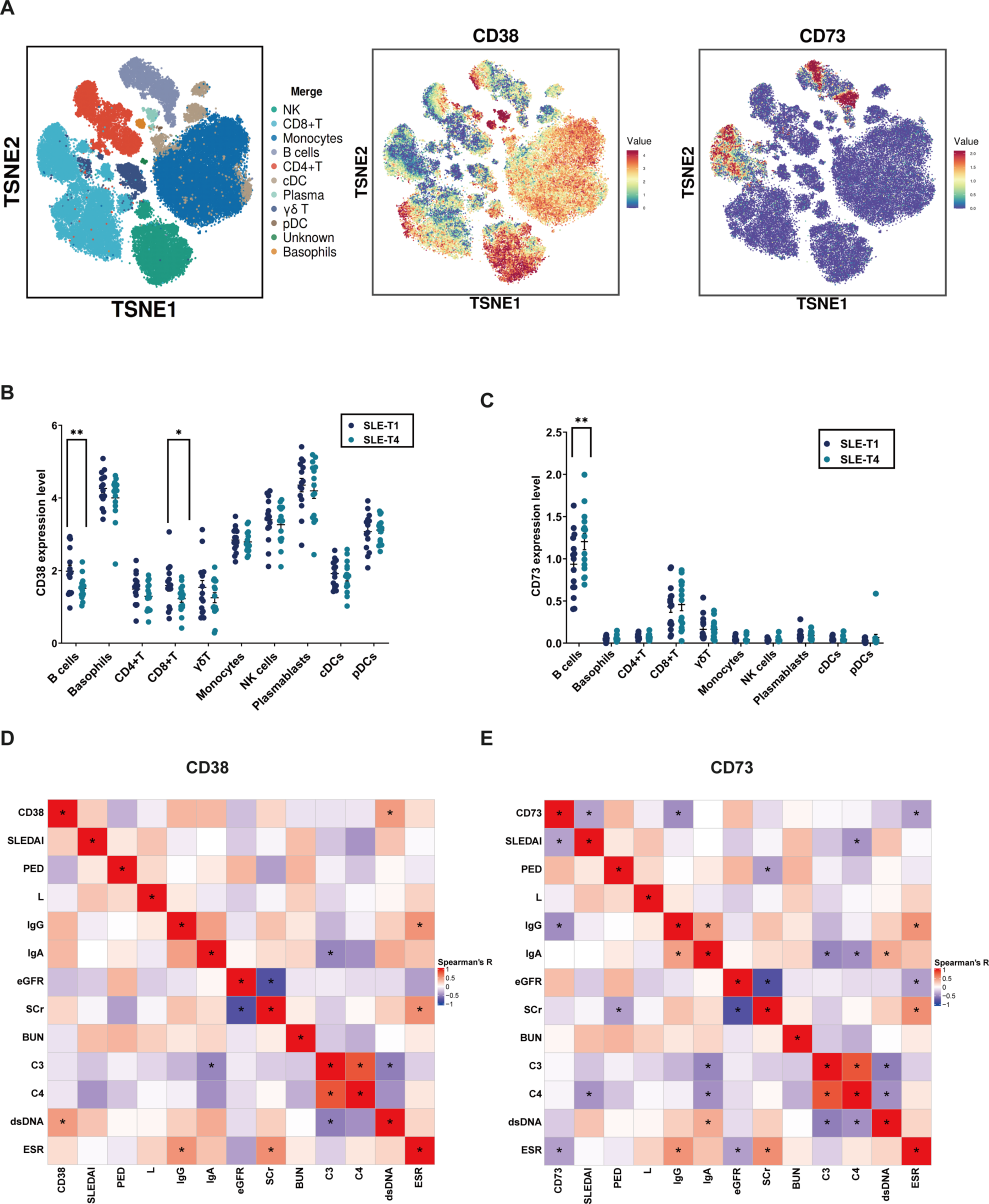
Expression profiling and correlation analysis of specific markers. **(A)** t-SNE visualization showing the expression of CD38 and CD73 across PBMCs. **(B)** Expression of CD38 in PBMC subpopulations. **(C)** Expression of CD73 in PBMC subpopulations. **(D)** Multivariate correlation analysis of B-cell CD38 expression with clinical indicators. **(E)** Multivariate correlation analysis of B-cell CD73 expression with clinical indicators. SLEDAI, Systemic Lupus Erythematosus Disease Activity Index; PED, Prednisone Equivalent Dose; L, Lymphocyte Count; eGFR, Estimated Glomerular Filtration Rate; Scr, Serum Creatinine; BUN, Blood Urea Nitrogen; C3, Complement Component 3; C4, Complement Component 4; dsDNA, Double-stranded DNA Antibodies; ESR, Erythrocyte Sedimentation Rate; pDC, Plasmacytoid Dendritic Cells; cDC, Conventional Dendritic Cells. Each symbol represents one subject. Color intensity indicates correlation strength (red, positive; blue, negative). Significance levels are indicated as: *P < 0.05, **P < 0.01.

T-SNE visualization of PBMCs revealed distinct expression patterns of CD38 and CD73 across immune cell subsets (**Figure 4A**). CD38 expression was highest in plasmablasts, followed by basophils, NK cells, and pDCs, whereas other immune cells exhibited lower levels (**Figure 4B**). Paired comparisons between SLE-T1 and SLE-T4 showed a significant decrease in CD38 expression in B cells and CD8^+^ T-cell subsets, with the reduction more pronounced in B cells (P < 0.01). Correlation analysis revealed that B-cell CD38 expression was positively associated with anti-dsDNA antibody levels (**Figure 4D**).

In contrast, CD73 expression was selectively upregulated in B cells (**Figure 4C**). Paired analysis showed higher CD73 expression in SLE-T4 compared to SLE-T1, suggesting an upregulation trend during disease recovery. Correlation analysis further indicated inverse associations between CD73 expression and SLEDAI scores, IgG levels, and ESR (**Figure 4E**).

### 3.6 Immune profiling of SLE-SR

**Figure 5:**
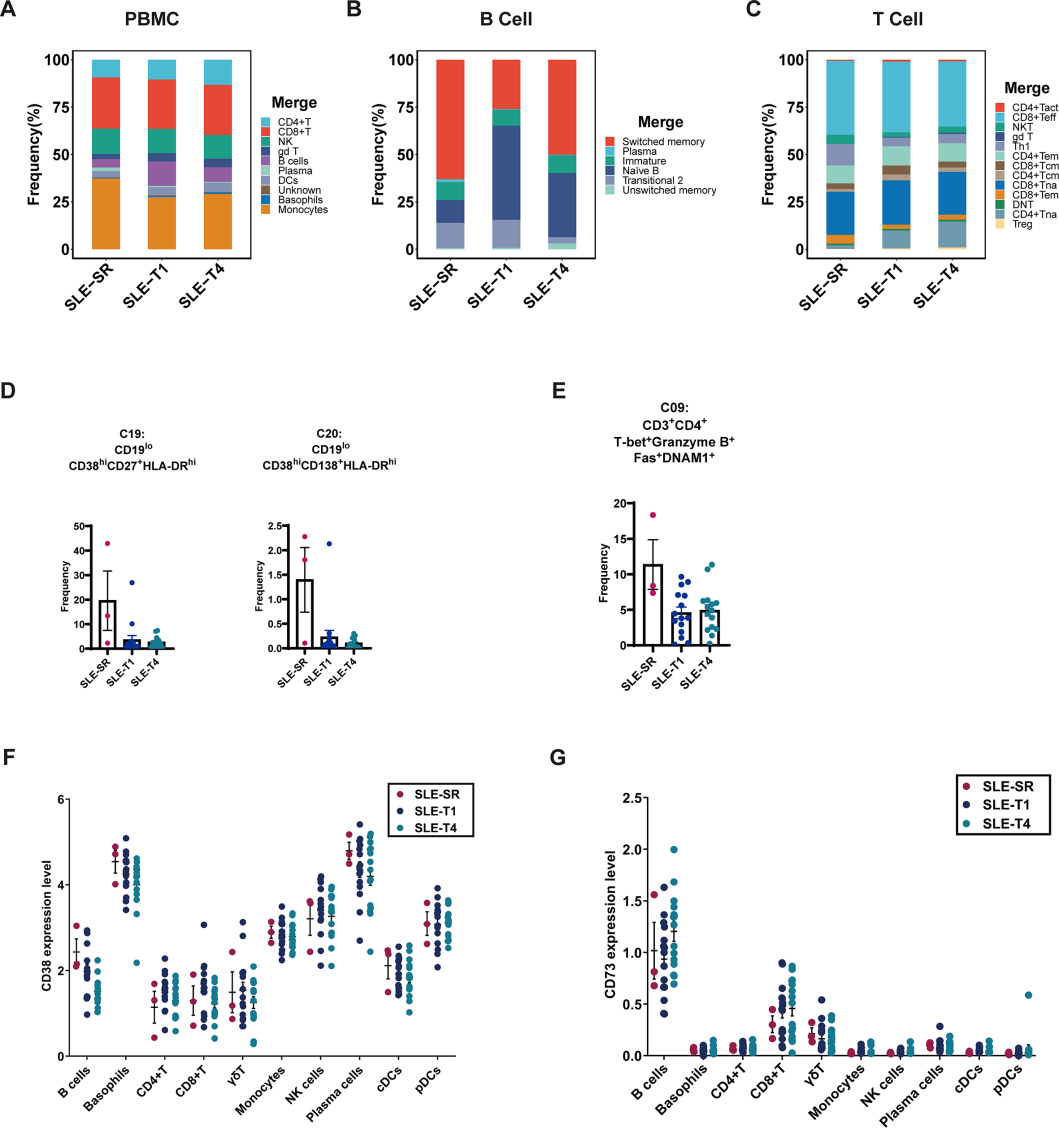
Immune profiling of severe refractory SLE (SLE-SR). **(A)** Stacked bar plot showing the distribution of major PBMC immune cell populations across SLE-T1, SLE-T4, and SLE-SR. **(B)** Stacked bar plot depicting the distribution of B-cell subpopulations across SLE-T1, SLE-T4, and SLE-SR. **(C)** Stacked bar plot depicting the distribution of T-cell subpopulations across SLE-T1, SLE-T4, and SLE-SR. **(D)** Frequencies of C19 (plasmablast) and C20 (plasma cell) subclusters within the B-cell compartment in SLE-T1, SLE-T4, and SLE-SR. **(E)** Frequency of the cytotoxic Th1-like CD4^+^ T-cell subcluster (C09) across the three groups. **(F)** CD38 expression across immune cell populations in SLE-T1, SLE-T4, and SLE-SR. **(G)** CD73 expression across immune cell populations in SLE-T1, SLE-T4, and SLE-SR. Fas, Fas Receptor (CD95); T-bet, T-box transcription factor TBX21; DNAM-1, DNAX Accessory Molecule-1. Each symbol represents one subject. Error bars represent SEM.

To provide a reference immune profile for refractory SLE, we first analyzed the overall composition of PBMCs in the SLE-SR cohort. Compared to the SLE-T1 and SLE-T4 cohorts, SLE-SR patients showed a relative reduction in overall B-cell frequency, accompanied by an increased proportion of plasma cells. Monocyte frequencies were also elevated in SLE-SR (**Figure 5A**).

Further B-cell subset analysis revealed a lower proportion of naïve B cells, along with an increased frequency of plasma cells and switched memory B cells (**Figure 5B****).** Re-clustering analysis also indicated an expansion of plasmablasts (C19: CD19^lo^CD38^hi^CD27^+^HLA-DR^hi^) and plasma cells (C20: CD19^lo^CD38^hi^CD138^+^HLA-DR^hi^) in SLE-SR patients (**Figure 5D**).

Analysis of T-cell populations showed a decrease in naïve CD4^+^ T cells, with a relative increase in Th1 cells in SLE-SR (**Figure 5C**). An expanded C09 subcluster (CD3^+^CD4^+^T-bet^+^Granzyme B^+^Fas^+^DNAM1^+^) was also observed in these patients (**Figure 5E****).** The presence of T-bet, a key transcription factor for Th1 differentiation, and Granzyme B, a marker of cytotoxic activity, suggests that this subcluster exhibits a strong Th1-like and cytotoxic phenotype.

We further examined CD38 and CD73 expression in SLE-SR. CD38 was broadly expressed across immune cells, with a trend toward higher expression in B cells compared to SLE-T1 and SLE-T4 (**Figure 5F****).** CD73 expression was selectively upregulated in B cells, with the highest levels observed in SLE-T4 (average SLEDAI score 3.5), followed by SLE-SR (average SLEDAI score 11.3), and the lowest levels in SLE-T1 (average SLEDAI score 15.1) (**Figure 5G****).** This descriptive observation suggests a potential inverse relationship between CD73 expression and disease activity, with higher levels during recovery and lower levels during active disease.

## 4 Discussion

The conventional approach to treating SLE primarily relies on broad-spectrum anti-inflammatory and immunosuppressive medications, such as glucocorticoids, antimalarials, and immunosuppressants. These agents are used either as monotherapy or combined, have historically served as the standard therapy for SLE. Despite significant advancements in diagnosing and managing SLE in recent years, several key challenges remain, including low remission rates, frequent relapses, and the risk of organ damage. These challenges are particularly pronounced in children with SLE (cSLE). Some cSLE patients do not achieve clinical remission even after prolonged treatment and may have risk for severe organ damage. Therefore, there is an imperative need to explore new therapeutic targets to improve patient outcomes.

The emergence of biologic therapies has provided new avenues for the treatment of SLE. Belimumab, a monoclonal antibody targeting BLyS, also known as B cell activating factor, was approved by the U.S. FDA in 2011 and subsequently in China in 2019 for adult SLE treatment. Its indications have since been expanded to include pediatric SLE and adult lupus nephritis (9, 18). Multiple clinical studies have demonstrated that combining belimumab with conventional therapy offers significant benefits for both adult and pediatric SLE patients, including reduced disease activity, slower progression of organ damage, and faster corticosteroid tapering, consistent with the outcomes observed in our cSLE cohort (7, 9, 19). Recent trials in patients with active lupus nephritis also suggest that belimumab may enhance induction therapy when combined with mycophenolate mofetil, highlighting its potential as an adjunctive treatment option (20). Concurrently, several studies have characterized the immunological effects of belimumab in adult SLE. Malmström et al. reported that in a prospective cohort of 23 adult SLE patients, belimumab treatment led to rapid and sustained reductions in naïve and CD11c^+^CD21^−^ B cells, while switched memory B cells and plasma cells remained stable over time (21). Similarly, Georg Pongratz et al. observed significant reductions in naïve and transitional B cells by month 6, which correlated with declining BLyS levels, along with a sustained increase in switched memory B cells at both month 6 and month 12. That study also found that baseline frequencies of CD8^+^ effector memory T cells were positively associated with baseline disease activity (22). Extending these findings to cSLE, our analysis of 15 patients treated with belimumab for 3 months revealed a parallel B-cell shift: marked reductions in naïve and transitional B cells and a relative increase in memory B cells—consistent with inhibition of BLyS signaling through its primary receptor BR3. We also observed a reduction in age-associated B cells (ABCs), a subset implicated in autoimmunity and chronic inflammation, suggesting that belimumab may modulate additional pathogenic B-cell populations in children (17). In the SLE-SR cohort, plasmablasts and plasma cells were relatively expanded, in some cases comprising over 40% of total B cells. Although the small sample size precludes statistical inference, this pattern is consistent with features of refractory disease and supports further consideration of plasma cell targeted strategies.

CD38 is an ectoenzyme glycoprotein involved in cell adhesion and signal transduction and is highly expressed on antibody-producing cells, particularly plasmablasts and plasma cells (23). Consistent with prior *in vitro* studies in SLE (24), we observed high CD38 expression on plasmablasts, with B-cell CD38 levels appearing highest in the SLE-SR group and showing trends aligned with disease activity and anti-dsDNA antibody titers. Long-lived plasma cells reside in specialized survival niches within bone marrow or inflamed tissues and are largely resistant to conventional immunosuppression and B-cell depletion, enabling persistent autoantibody production and contributing to refractory disease (25–27). Given the high expression of CD38 on these cells, CD38-targeted therapies represent a mechanistically plausible strategy in refractory SLE. Monoclonal antibodies such as daratumumab and mezagitamab have shown potential efficacy (28–30). A case report described two patients with severe, life-threatening refractory SLE who achieved clinical improvement following daratumumab, accompanied by reductions in autoantibody levels, circulating plasmablasts, and type I interferon activity (29). Small case series in refractory lupus nephritis also reported clinical improvement and good tolerability with daratumumab monotherapy (30). Beyond SLE, daratumumab has demonstrated benefits in other autoantibody-driven diseases, including primary Sjögren’s syndrome, ANCA-associated vasculitis, and refractory immune thrombocytopenia (31). Collectively, these findings support a role for CD38 in autoimmune pathophysiology and justify further exploration of CD38-targeted therapies. However, given the broad expression of CD38 across immune cell populations, potential risks such as increased infection susceptibility and disruption of lymphocyte homeostasis warrant careful evaluation in future studies.

CD73, in contrast, is preferentially expressed on B cells. In our cohort of cSLE patients, CD73 expression on B cells was inversely correlated with disease activity, with higher CD73 levels associated with lower disease burden. CD73 (ecto-5’-nucleotidase) is a GPI-anchored enzyme that works together with CD39 to hydrolyze AMP into adenosine, which possesses anti-inflammatory properties and plays a key role in regulating immune responses (32). Previous studies have shown that B cell subpopulations with high CD73 expression can exert immunosuppressive effects via adenosine production, independent of IL-10 (33). In SLE mouse models, deficiency of CD39 and CD73 leads to increased autoantibody production, splenic expansion of B and T cells, elevated plasma-free DNA, and endothelial dysfunction (34). Similarly, in adult SLE patients, the proportion of CD73^+^ B cells is reduced, particularly within the CD39^+^CD73^+^ subset, accompanied by decreased serum adenosine levels; these alterations correlate with disease activity and laboratory parameters (35). Taken together, our findings and existing evidence support a potential modulatory role for CD73 in SLE pathogenesis and highlight the CD73-mediated adenosine signaling pathway as a promising avenue for further investigation.

Beyond B-cell alterations, we identified a cytotoxic Th1-like CD4^+^ T cell subcluster (C09) in severe, refractory SLE. While CD4 CTLs are normally a minor effector subset, their proportion is elevated in autoimmune diseases such as IgG4-RD, primary Sjögren’s syndrome, and systemic sclerosis (36, 37). In our study, this expansion was observed specifically in refractory cSLE, suggesting a potential link to disease resistance or organ involvement, though whether it represents a treatment-induced or pathogenic subset remains unclear.

In summary, this study characterizes immunophenotypic changes in cSLE, highlighting pronounced B-cell alterations during belimumab treatment. In refractory cSLE, the persistence of plasma cells and plasmablasts may underlie incomplete responses, suggesting CD38 as a potential therapeutic target. CD73 expression patterns point to a modulatory role of the adenosine pathway in SLE. Furthermore, a cytotoxic Th1-like CD4^+^ T cell subcluster was observed in severe SLE, revealing an underexplored aspect of disease pathogenesis.

### 4.1 Study Limitations

Several limitations should be acknowledged. The sample size was small, particularly for the SLE-SR cohort, which served only as a descriptive reference. Although the longitudinal cohort was within-subject, heterogeneity in disease duration, organ involvement, and prior therapy may have influenced immune phenotypes. CyTOF analysis is observational and descriptive, allowing characterization of immune subsets but not causal inference. Finally, despite correction for multiple testing, correlation-based findings should be interpreted cautiously and require validation in larger, independent cohorts.

## Data Availability

All data necessary to support the findings of this study are included in the article, figures, and supplementary materials. Additional data are available from the corresponding author upon reasonable request.

## Supplementary material

Supplementary Figure 1 Features of CD3^-^CD19^-^ immune cell subsets in cSLE patients. **(A, B)** t-SNE visualization and phenotype annotation of CD3^−^CD19^−^ immune cells, showing their distribution and clustering, with each cluster annotated by the corresponding cell phenotype. **(C)** Heatmap summarizing the relationships between CD3^−^CD19^−^ immune cell clusters and marker expression patterns. **(D)** Changes in CD3^−^CD19^−^ immune cell subpopulation frequencies. Left: descriptive comparison of cell subpopulation distributions across SLE-T1, SLE-T4, and SLE-SR. Right: paired comparison of cell subpopulation frequencies before and after belimumab treatment in cSLE patients. NK, Natural Killer Cells; pDC, Plasmacytoid Dendritic Cells; cDC, Conventional Dendritic Cells. Error bars represent SEM.

Supplementary Table 1 The antibody reagents used in CyTOF.
