# Supplementary material for: CyTOF analysis of immune characteristics in cSLE: belimumab treatment and refractory cases
Source: Front Immunol. 2026 Feb 2;17:1699104. doi: 10.3389/fimmu.2026.1699104 (PMC12907313; doi:10.3389/fimmu.2026.1699104)
Supplement: Supplementary Figure 1 — Features of CD3-CD19- immune cell subsets in cSLE patients. (A, B) t-SNE visualization and phenotype annotation of CD3−CD19− immune cells, showing their distribution and clustering, with each cluster annotated by the corresponding cell phenotype. (C) Heatmap summarizing the relationships between CD3−CD19− immune cell clusters and marker expression patterns. (D) Changes in CD3−CD19− immune cell subpopulation frequencies. Left: descriptive comparison of cell subpopulation distributions across SLE-T1, SLE-T4, and SLE-SR. Right: paired comparison of cell subpopulation frequencies before and after belimumab treatment in cSLE patients. NK, Natural Killer Cells; pDC, Plasmacytoid Dendritic Cells; cDC, Conventional Dendritic Cells. Error bars represent SEM. [file Image1.pdf]

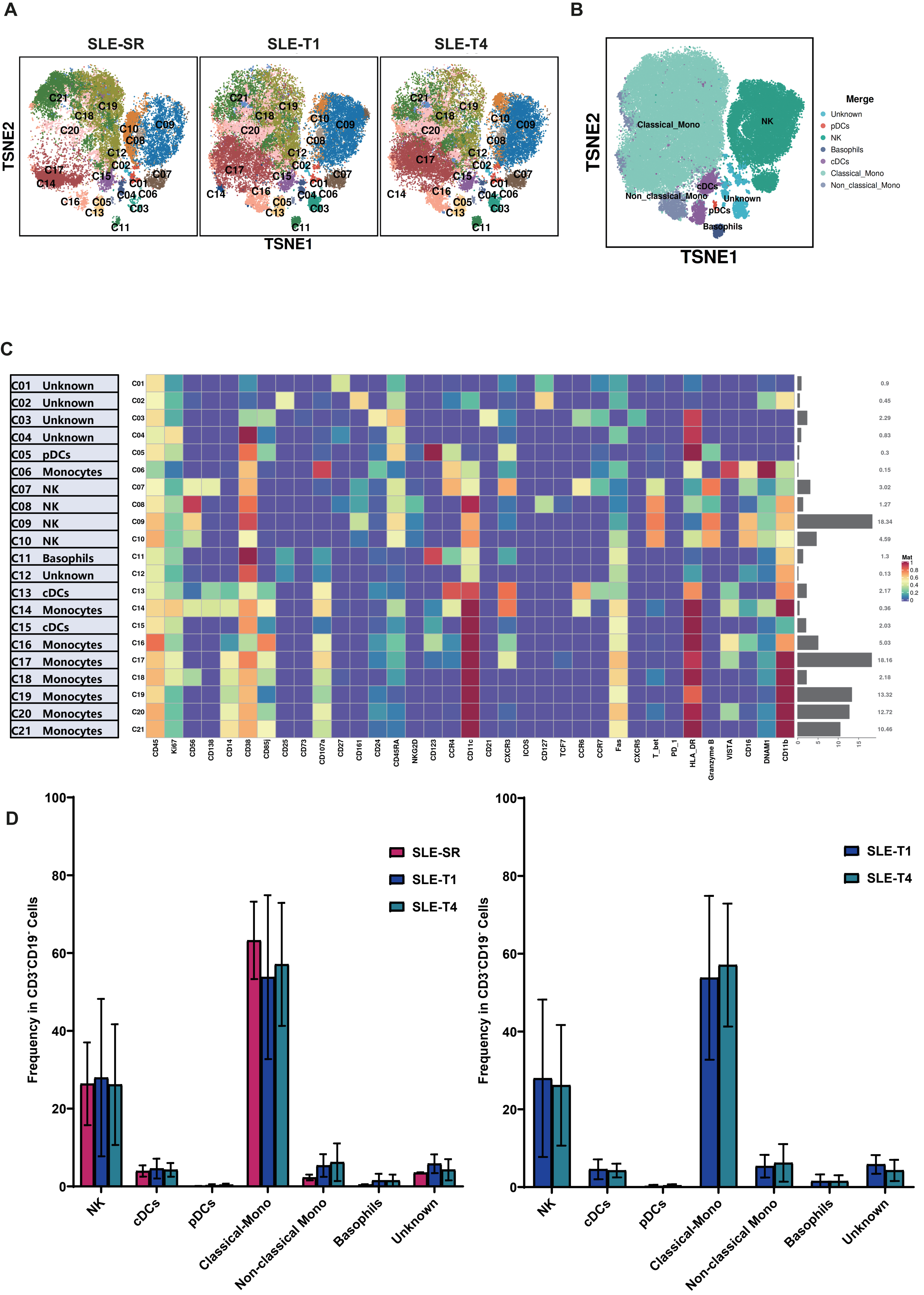
A
SLE-SR
SLE-T1
SLE-T4
TSNE2
TSNE1
C01 C02 C03 C04 C05 C06 C07 C08 C09 C10 C11 C12 C13 C14 C15 C16 C17 C18 C19 C20 C21
B
TSNE2
TSNE1
Classical_Mono
NK
cDCs
Non_classical_Mono
Unknown
pDCs
Basophils
Merge
Unknown
pDCs
NK
Basophils
cDCs
Classical_Mono
Non_classical_Mono
C
C01 Unknown
C02 Unknown
C03 Unknown
C04 Unknown
C05 pDCs
C06 Monocytes
C07 NK
C08 NK
C09 NK
C10 NK
C11 Basophils
C12 Unknown
C13 cDCs
C14 Monocytes
C15 cDCs
C16 Monocytes
C17 Monocytes
C18 Monocytes
C19 Monocytes
C20 Monocytes
C21 Monocytes
CD45 Ki67 CD56 CD138 CD14 CD38 CD85j CD25 CD73 CD107a CD27 CD161 CD24 CD45RA NKG2D CD123 CCR4 CD11c CD21 CXCR3 ICOS CD127 TCF7 CCR6 CCR7 Fas CXCR5 T_bet PD_1 HLA_DR Granzyme B VISTA CD16 DNAM1 CD11b
0.9
0.45
2.29
0.83
0.3
0.15
3.02
1.27
18.34
4.59
1.3
0.13
2.17
0.36
2.03
5.03
18.16
2.18
13.32
12.72
10.46
Mat
D
Frequency in CD3⁻CD19⁻ Cells
SLE-SR
SLE-T1
SLE-T4
NK
cDCs
pDCs
Classical-Mono
Non-classical Mono
Basophils
Unknown

Fig. S1. Features of CD3⁻CD19⁻ immune cell subsets in cSLE patients. A-B. t-SNE visualization and phenotype annotation of CD3⁻CD19⁻ immune cells, showing their distribution and clustering, with each cluster annotated by the corresponding cell phenotype. **C.** Heatmap summarizing the relationships between CD3⁻CD19⁻ immune cell clusters and marker expression patterns. **D.** Changes in CD3⁻CD19⁻ immune cell subpopulation frequencies. **Left:** descriptive comparison of cell subpopulation distributions across SLE-T1, SLE-T4, and SLE-SR. **Right:** paired comparison of cell subpopulation frequencies before and after belimumab treatment in cSLE patients. NK, Natural Killer Cells; pDCc, Plasmacytoid Dendritic Cells; cDC, Conventional Dendritic Cells. Error bars represent SEM.
