# Supplementary material for: CyTOF analysis of immune characteristics in cSLE: belimumab treatment and refractory cases
Source: Front Immunol. 2026 Feb 2;17:1699104. doi: 10.3389/fimmu.2026.1699104 (PMC12907313; doi:10.3389/fimmu.2026.1699104)
Supplement: Supplementary Table 1 — The antibody reagents used in CyTOF. [file Table1.pdf]

Table S1 The antibody reagents used in CyTOF.

List	Metal channels	Antibody Names	List	Metal channels	Antibody Names	List	Metal channels	Antibody Names	List	Metal channels	Antibody Names
1	89Y	CD45	12	148Nd	TCR γ/δ	23	159Tb	CD11c	34	170Er	T-bet
2	115In	CD3	13	149Sm	CD25	24	160Gd	CD21	35	171Yb	CD279(PD-1)
3	139La	Ki67	14	150Nd	CD73	25	161Dy	CD183(CXCR3)	36	172Yb	HLA-DR
4	141Pr	CD56	15	151Eu	CD107a	26	162Dy	CD278(ICOS)	37	173Yb	Granzyme B Recombinant
5	142Nd	CD138	16	152Sm	CD27	27	163Dy	CD127(IL-7Rα)	38	174Yb	VISTA(B7-H5,PD-1H)
6	143Nd	IgD	17	153Eu	CD161	28	164Dy	TCF1(TCF7)	39	175Lu	CD16
7	144Nd	CD14	18	154Sm	CD24	29	165Ho	CD66b	40	176Yb	CD226(DNAM1)
8	145Nd	CD20	19	155Gd	CD45RA	30	166Er	CD196(CCR6)	41	197Au	CD4
9	146Nd	CD38	20	156Gd	CD314(NKG2D)	31	167Er	CD197(CCR7)	42	198Pt	CD8a
10	147Sm	CD85j(ILT2)	21	157Gd	CD123(IL-3Rα)	32	168Er	CD95(Fas)	43	209Bi	CD11b
11	148Nd	CD19	22	158Gd	CD194(CCR4）	33	169Tm	CD185(CXCR5)			
