# Supplementary material for: CyTOF analysis of immune characteristics in cSLE: belimumab treatment and refractory cases
Source: Front Immunol. 2026 Feb 2;17:1699104. doi: 10.3389/fimmu.2026.1699104 (PMC12907313; doi:10.3389/fimmu.2026.1699104)
Supplement: Supplementary file 4 [file DataSheet2.pdf]

Supplementary Methods:

CyTOF Analysis

PBMC Isolation and Preparation

Peripheral blood mononuclear cells (PBMCs) were isolated from peripheral blood using density gradient centrifugation. Cells were counted, and 1×10^6 cells per sample were used for downstream CyTOF staining and analysis.

Viability Staining and Fc Receptor Blocking

Cells were resuspended in FACS buffer containing 250 nM cisplatin (Cell-ID Cisplatin-194Pt, Fluidigm) to assess viability. To reduce non-specific binding, cells were treated with an Fc receptor–blocking solution (BioLegend) following the manufacturer's instructions.

Surface and Intracellular Staining

Surface markers were stained with a pre-mixed extracellular antibody cocktail on ice for 30 minutes. Following surface staining, cells were fixed and permeabilized using intercalation/fixation buffer (Fluidigm) for intracellular marker staining. Mass-tag cellular barcoding (MCB) was applied to minimize variability in staining efficiency and instrument sensitivity. Details of all 43 metal-conjugated antibodies, including target antigen, clone, and metal tag, are provided in Supplementary Table S1.

CyTOF Data Acquisition

Data acquisition was performed on a Helios CyTOF system (Fluidigm), calibrated using tuning solution and EQ Four Element Calibration Beads. Cells were diluted to 1×10^6 cells/mL in deionized water containing 20% EQ beads and filtered prior to acquisition. Over 1 million events were recorded per sample. Acquisition order was randomized to minimize batch effects, and raw FCS files were normalized using bead-based normalization.

Data Preprocessing

Preprocessing included removal of debris, doublets, and dead cells based on event length, DNA intercalator (191Ir/193Ir), and cisplatin (194Pt) signals. For visualization, 30,000 cells were randomly downsampled from each donor sample to ensure comparable representation across donors.

Dimensionality Reduction and Clustering

Dimensionality reduction was performed using t-SNE with a perplexity of 100 and 1000 iterations, implemented in the R package cytofkit. Unsupervised clustering was conducted using the PhenoGraph algorithm, with k = 30 nearest neighbors and Euclidean distance for graph construction. The number of clusters was determined automatically by community detection in the k-nearest neighbor graph. Hierarchical clustering of median marker expression was applied to assess cluster consistency across samples.

Cluster Annotation

Clusters were annotated based on canonical lineage markers and subset-specific markers. Major immune cell populations were identified. Detailed gating strategies and marker definitions for each immune cell subset are provided in the supplementary Excel table (Supplementary_Table_Immune_Cell_Subsets_CyTOF.xlsx). Clusters are also indicated alongside the heatmaps to facilitate interpretation.

Reproducibility Measures

To ensure reproducibility, standardized antibody panels, staining protocols, and CyTOF acquisition settings were applied across all samples. Instrument performance was continuously

monitored using EQ beads, and hierarchical clustering of median marker expression was used consistently to validate cell population identification across donors.
